# Pathophysiological Roles of Neuro-Immune Interactions between Enteric Neurons and Mucosal Mast Cells in the Gut of Food Allergy Mice

**DOI:** 10.3390/cells10071586

**Published:** 2021-06-23

**Authors:** Tomoe Yashiro, Hanako Ogata, Syed Faisal Zaidi, Jaemin Lee, Shusaku Hayashi, Takeshi Yamamoto, Makoto Kadowaki

**Affiliations:** 1 Division of Gastrointestinal Pathophysiology, Institute of Natural Medicine, University of Toyama, 2630 Sugitani, Toyama 930-0194, Japan; tomoe.yashiro@med.tonami.toyama.jp (T.Y.); d1761302@ems.u-toyama.ac.jp (H.O.); sfaisalhz@gmail.com (S.F.Z.); plzhug@gmail.com (J.L.); hayashi@inm.u-toyama.ac.jp (S.H.); ty@inm.u-toyama.ac.jp (T.Y.); 2 Department of Pharmacology, School of Medicine, Batterjee Medical College for Sciences and Technology, Jeddah 21442, Saudi Arabia

**Keywords:** mucosal mast cell, enteric neuron, adenosine, FcεRI, neuro-immune interaction, food allergy

## Abstract

Recently, the involvement of the nervous system in the pathology of allergic diseases has attracted increasing interest. However, the precise pathophysiological role of enteric neurons in food allergies has not been elucidated. We report the presence of functional high-affinity IgE receptors (FcεRIs) in enteric neurons. FcεRI immunoreactivities were observed in approximately 70% of cholinergic myenteric neurons from choline acetyltransferase-eGFP mice. Furthermore, stimulation by IgE-antigen elevated intracellular Ca^2+^ concentration in isolated myenteric neurons from normal mice, suggesting that FcεRIs are capable of activating myenteric neurons. Additionally, the morphological investigation revealed that the majority of mucosal mast cells were in close proximity to enteric nerve fibers in the colonic mucosa of food allergy mice. Next, using a newly developed coculture system of isolated myenteric neurons and mucosal-type bone-marrow-derived mast cells (mBMMCs) with a calcium imaging system, we demonstrated that the stimulation of isolated myenteric neurons by veratridine caused the activation of mBMMCs, which was suppressed by the adenosine A3 receptor antagonist MRE 3008F20. Moreover, the expression of the adenosine A3 receptor gene was detected in mBMMCs. Therefore, in conclusion, it is suggested that, through interaction with mucosal mast cells, IgE-antigen-activated myenteric neurons play a pathological role in further exacerbating the pathology of food allergy.

## 1. Introduction

The number of patients with food allergies (FAs) has significantly increased in recent decades. Despite the increasing prevalence of FAs, the pathogenic mechanisms underlying FAs are not fully understood. Thus, the therapeutic options remain limited, and useful drug therapies against FAs are not available [1].

It is well known that mast cells are primarily involved in the pathology of various allergic diseases. In particular, in immediate allergic reactions, the onset of allergic symptoms is mainly associated with the release of proinflammatory chemical mediators from mast cells by IgE-antigen stimulation via high-affinity IgE receptors (FcεRIs) on mast cells. We previously demonstrated that the number of mucosal mast cells (MMCs) is greatly increased in the colonic mucosa of ovalbumin (OVA)-induced food allergy (FA) mice, and MMCs, but not connective tissue mast cells (CTMCs), play the most pivotal role in the pathology of FAs [2]. Mast cells are classified as MMCs or CTMCs. Considerable evidence has demonstrated that MMCs are morphologically, biochemically, and functionally distinct from CTMCs [3]. CTMCs are located within connective tissues, such as the skin, whereas MMC progenitors migrate to mucosal tissues, such as intestinal mucosa, respiratory mucosa, and vaginal mucosa, where MMCs are able to mature following some kind of immunological stimulation, such as cytokine exposure and antigen exposure. CTMCs contain high concentrations of histamine in their granules and mainly contribute to allergic symptoms, such as congestion, itching, urticaria, and anaphylaxis, whereas MMCs contain low concentrations of histamine [4]. Consequently, CTMC stabilizers (e.g., tranilast, ketotifen, and cromolyn) are frequently used for the treatment of various allergic disorders, while these stabilizers fail to exert therapeutic effects on FAs, which is supported by our finding that these stabilizers are not able to suppress MMC activation [5].

Mast cells can also be activated in response to ligands, such as substance P, leukotrienes, histamine, cytokines, ATP, adenosine, and complements mediated by G-protein-coupled receptors, and nonimmunological mast cell activation by these ligands is primarily associated with chronic inflammation, where they act in a paracrine or autocrine fashion to amplify or modulate mast cell function, and this mechanism accounts for mast cell involvement in various diseases [6,7].

Recent studies have highlighted the pathophysiological roles of the nervous system and neuro-immune interactions in the development of allergic diseases [8,9]. Several studies have demonstrated the cross-communication between neurons and mast cells, whereby they activate each other in allergic diseases [2,8,9,10,11]. Interestingly, the close communication between neurons and immune cells, particularly mast cells, predominantly occurs in barrier tissues, including the skin, respiratory tract, and gastrointestinal tract, which are exposed to allergens [8,9,10,11].

The enteric nervous system is one part of the autonomic nervous system, which regulates gastrointestinal functions, such as motor function and electrolyte/water secretion, independently of the central nervous system. The enteric nerve plexuses contain sensory neurons, interneurons, and motor neurons, thereby forming intrinsic neural networks in the intestine. All the neurons in the enteric nervous system have now been classified into at least 14 defined neuron types in terms of their morphologies, projections, primary neurotransmitters, neurochemical properties, and physiological functions [12]. Enteric neurons are able to secrete a variety of neurotransmitters, including acetylcholine (ACh), ATP, nitric oxide, serotonin, calcitonin-gene-related peptide (CGRP), and substance P. We previously reported a marked increase in MMCs, CGRP-immunoreactive cholinergic sensory nerve fibers and the close apposition between these MMCs and nerve fibers in the colon during the development of FAs [2,10,11]. Furthermore, we demonstrated the contribution of CGRP to the development of FAs due to the augmentation of microtubule reorganization in MMCs [11]. Sensory neurons are important for the initiation of allergic responses [8,9] as well as neural circuit activation, and the activation of sensory neurons has been suggested to be triggered by immunoglobulin–antigen immune complexes because the specific receptors for IgG and IgE are expressed on mouse dorsal root ganglion neurons [13,14]. Another brief report demonstrated that FcεRIs are present in mouse superior cervical ganglion (SCG) neurons and that IgE-antigen stimulation activates mouse SCG neurons and cells in the mouse myenteric ganglion [15]. However, to date, no detailed and precise report has demonstrated the expression of FcεRI on myenteric neurons or the activation of myenteric neurons by IgE-antigen stimulation.

While the enteric nervous system contains a variety of neurotransmitters and neuromodulators, purines, such as ATP and adenosine, participate in synaptic transmission as neurotransmitters or neuromodulators [16]. Adenosine is released from longitudinal muscle myenteric plexus preparations (LMMPs) of the guinea pig ileum upon electrical stimulation [17]. Kadowaki et al. demonstrated that adenosine could mediate intestinal relaxation through two different inhibitory receptor subtypes: adenosine A1 receptors on enteric neurons and adenosine A2b receptors on the smooth muscle in the guinea pig distal colon [18]. Purinergic signaling plays an important role in the regulation of enteric reflexes and overall gastrointestinal function [16]. Adenosine functions in an autocrine or paracrine fashion through binding to purinergic P1 receptors, namely adenosine A1, A2a, A2b, and A3 receptors that display distinct pharmacological characteristics. Adenosine has long been implicated in a variety of inflammatory processes, including allergy and especially asthma [6,7,19,20,21]. Bone-marrow-derived mast cells (BMMCs) express the adenosine A2a, A2b, and A3 receptor subtypes, and adenosine A3 receptors are highly abundant and play a predominant role in mediating hyperresponsiveness to adenosine in mast cells [6,7,22]. Adenosine A3 receptor signaling through Gi2, Gi3, or Gq proteins leads to adenylyl cyclase inhibition and phospholipase C stimulation and increased diacylglycerol, IP3, and intracellular calcium concentrations [6,7].

In this study, we aimed to elucidate the pathological crosstalk between enteric neurons and MMCs through adenosine A3 receptor signaling using an in vitro coculture system.

## 2. Methods

### 2.1. Mice

Male BALB/c mice were purchased from Japan SLC (Shizuoka, Japan), and choline acetyltransferase (ChAT)-enhanced green fluorescent protein (eGFP) transgenic mice (ChAT-eGFP mice: B6.Cg-Tg (RP23-268L19-EGFP)2Mik/J) were obtained from the Jackson Laboratory (Bar Harbor, ME, USA). The animal experiments were performed in accordance with the National Institutes of Health (NIH) guide for the care and use of laboratory animals. This study was approved by the Animal Experiment Committee at the University of Toyama (Authorization No. A2012inm-4 and A2015inm-3).

### 2.2. Food Allergy Model

FA induction was performed as previously described [2,10,11]. Briefly, 5-week-old male BALB/c mice were sensitized twice, with an intervening two-week interval, by intraperitoneal injection of 50 μg of OVA (fraction V; Sigma-Aldrich, St. Louis, MO, USA) in the presence of an aluminum hydroxide gel adjuvant (Thermo scientific, Rockford, IL, USA). Two weeks later, the mice were repeatedly given 50 mg of OVA every other day. Diarrhea was assessed by visually monitoring the mice for up to 1 h following the oral OVA challenge. The profuse liquid stool was detected as allergic diarrhea, and the diarrhea-presenting mice were considered to be FA mice. The occurrence of allergic diarrhea reached about 80.0% after the 6th OVA challenge. Tissue samples were collected 1 h after the 6th oral OVA challenge.

### 2.3. Immunohistochemistry

Immunohistochemistry was performed as described in our previous report [2,10]. The proximal colon was fixed with 4% paraformaldehyde (*w*/*v*) in 0.1 M sodium phosphate buffer (pH 7.3) and immersed for 12–18 h in the same fixative at 4 °C. The tissue was washed with 0.01 M phosphate-buffered saline (PBS; pH 7.3), cryoprotected with 30% sucrose in 0.01 M PBS, and embedded in optimal cutting tissue (OCT) compound. Frozen sections (30 μm) were cut at −20 °C using a cryostat microtome (Leica Microsystems, Nussloch, Germany). The sections were soaked for 12–18 h in 0.01 M PBS containing 0.3% Triton X-100 to increase permeability, exposed to normal donkey serum (1:10; Jackson ImmunoResearch Laboratories, West Grove, PA, USA) for 30 min to reduce the nonspecific binding of antisera, and washed in 0.01 M PBS. The sections were exposed to each primary antibody for 12–18 h, washed with 0.01 M PBS and incubated with the appropriate secondary antibody for 2 h. The sections were rinsed in 0.01 M PBS and mounted in a mounting medium, including DAPI (Vector Laboratories, Peterborough, UK). The preparations were observed using confocal laser-scanning microscopy (LSM700; Carl Zeiss Japan, Tokyo, Japan). The following primary antibodies were used: sheep anti-mouse mast cell protease (mMCP)-1, a marker of mouse mucosal mast cells (1:5000; Moredun Scientific, Scotland, UK), and rabbit anti-neuronal β3-tubulin, a marker of neurons (1:10,000; Covance, Princeton, NJ, USA). Cy3-conjugated donkey anti-sheep IgG (1:200; Jackson ImmunoResearch Laboratories) and Cy3-conjugated donkey anti-rabbit IgG (1:200; Jackson ImmunoResearch Laboratories) were used as secondary antibodies. mMCP-1 immunoreactive areas were quantitatively analyzed in square areas (0.01 mm^2^) of colonic mucosa using ImageJ software (NIH, Bethesda, MD, USA). Five randomly selected areas were evaluated in at least three preparations from three animals.

For whole-mount preparations, LMMPs were detached from the small intestine of ChAT-eGFP mice. They were fixed in 4% paraformaldehyde and then permeabilized with 0.3% Triton X-100. These preparations were incubated with goat anti-α-subunit of FcεRI (FcεRIα) antibody (1:200; Santa Cruz Biotechnology, Dallas, TX, USA) as a primary antibody for 12–18 h at 4 °C, washed with 0.01 M PBS, and incubated with Cy3-donkey anti-goat IgG (1:400, Jackson ImmunoResearch) for 2 h at room temperature. These preparations were observed by a confocal laser scanning microscope (LSM700). In the present study, we selected one representative ganglion from each of five mice, and then these immunostained ganglia were quantitatively analyzed by ImageJ software (NIH, Bethesda, MD, USA).

### 2.4. Isolation of Myenteric Neurons

LMMPs were detached from the small intestine of 4–6-week-old BALB/c or ChAT-eGFP mice. LMMPs from normal BALB/c mice were incubated with 4500 U of collagenase type 2 (Worthington Biochemical Corporation, Lakewood, NJ, USA) in 10 mL of calcium-free Kreb’s solution (121.03 mM NaCl, 5.95 mM KCl, 14.30 mM NaHCO_3_, 1.34 mM NaH_2_PO_4_, 1.20 mM MgCl_2_, 2.50 mM CaCl_2_, 12.70 mM glucose; pH 7.4) for 1 h at 37 °C. The suspension was centrifuged at 300*g* for 5 min. Then, the pellet was suspended in the plating medium (DMEM/F12 containing 10% FBS) and plated on 12 mm glass-bottom dishes coated with 20 µg/mL poly-L-lysine Hbr (Sigma-Aldrich) and 10% Matrigel (BD Biosciences, San Jose, CA, USA). After 6 h, the plating medium was exchanged with feeding medium: neurobasal-A Medium (Thermo Fisher Scientific, Waltham, MA, USA) containing 20 ng/mL EGF, 20 ng/mL bFGF, 10 ng/mL βNGF, 10 mL/L antibiotic/antimycotic solution, and 0.1 mg/mL L-glutamine. Thereafter, the feeding medium was changed every other day. Cytarabine (10 µM, Ara-C: Sigma-Aldrich) was added to the medium from day 2 to 6. These cells were cultured for at least 8 days.

The cultures of enzyme-dissociated LMMP cells initially comprised several types of cell populations. However, after 4 days in culture, myenteric neurons started to dominate the culture, and these cell bodies with axons, along with several neurites, appeared in the culture dish. These myenteric neurons were observed by a microscope (CKX41; OLYMPUS, Tokyo, Japan) with the Hoffman Modulation Contrast system.

To further verify the successful in vitro culturing of myenteric neurons, we utilized the LMMPs of ChAT-eGFP mice and cultured myenteric neurons in conditions similar to those mentioned above.

### 2.5. Calcium Imaging in Cultured Myenteric Neurons

Cultured myenteric neurons were sensitized by incubation with 1.5 µg of monoclonal mouse anti-2,4-dinitrophenol (DNP) IgE (Yamasa, Tokyo, Japan) for 6 h. The cells were washed with Kreb’s solution and then loaded with 3 µM Fluo-8 AM (AAT Bioquest, Sunnyvale, CA, USA) for 30 min. After washing with Kreb’s solution, intracellular Ca^2+^ concentration ([Ca^2+^]i) was determined by measuring the fluorescence intensity for 500 s by a microscope (IX71; OLYMPUS) with a high-speed and high-sensitivity imaging system (MiCAM02; Brainvision, Tokyo, Japan). DNP-BSA (100 ng/mL) was added to the dish at 20 s, and A23187 (10 µM) was added to the dish at 250 s after starting the measurement for maximum stimulation of myenteric neurons. The activation rate was calculated by the following formula: (highest fluorescence intensity from 20 to 249 s/highest fluorescence intensity from 250 to 500 s) × 100.

### 2.6. Mucosal-Type Bone-Marrow-Derived Mast Cells (mBMMC)

We prepared mBMMCs from a 6-week-old male mouse (BALB/c) according to a previously described method [23]. Briefly, bone marrow cells were cultured in RPMI-1640 medium (Sigma-Aldrich) supplemented with 10% heat-inactivated fetal calf serum (FCS) (JRH Biosciences, Lenexa, KS, USA), 10 μM 2-mercaptoethanol (Wako, Osaka, Japan), 20 mM Hepes buffer (Sigma-Aldrich), 1 mM sodium pyruvate (Sigma-Aldrich), 100 μM MEM nonessential amino acids (Sigma-Aldrich), 2 μg/mL gentamicin solution (Sigma-Aldrich), 20 μL/mL stabilized penicillin–streptomycin solution (Sigma-Aldrich), 20 ng/mL recombinant murine interleukin-3 (IL-3; Peprotech, Rocky Hill, NJ, USA), 40 ng/mL recombinant murine SCF (Peprotech), 5 ng/mL recombinant murine IL-9 (R&D systems, Minneapolis, MN, USA), and 1 ng/mL TGF-β1 (Sigma-Aldrich) at 37 °C in a humidified 5% CO_2_ atmosphere. Mast cell purity was examined by flow cytometry (FACSCalibur; Becton Dickinson, Franklin Lakes, NJ, USA), and more than 98% of the nonadherent cells were positive for FcεRI and c-kit.

### 2.7. Calcium Imaging in mBMMCs Co-Cultured with Isolated Myenteric Neurons

mBMMCs were loaded with 3 µM Fluo-8 AM (AAT Bioquest) for 30 min. After washing with Kreb’s solution, mBMMCs were added to a dish of isolated myenteric neurons for an enteric neuron–immune cell coculture system. Then, [Ca^2+^]i in mBMMCs was measured as fluorescence intensity with MiCAM02. Neurostimulator veratridine (Na^+^ channel activator; 10 µM; Sigma-Aldrich), for stimulation of only isolated myenteric neurons, was added to a dish at 20 s, and calcium ionophore A23187 (10 µM) was added to the dish at 250 s after starting the measurement for maximum stimulation of mBMMCs. The activation rate was calculated by the following formula: (the highest fluorescent intensity from 20 s to 249 s/the highest fluorescent intensity from 250 s to 500 s) × 100. Selective adenosine A3 receptor antagonist MRE 3008F20 (R&D systems) at 10 μM was applied to the dish 20 min before the administration of veratridine.

### 2.8. Measurement of Intracellular Calcium Concentration in mBMMC

Sensitized mBMMCs with mouse anti-DNP IgE were loaded with 5 μM Fura-2 AM (Dojindo, Kumamoto, Japan) in loading buffer (118 mM NaCl, 4.7 mM KCl, 1 mM Na_2_HPO_4_, 1.13 mM MgCl_2_, 10 mM HEPES, 5.5 mM D-glucose, 100 mM L-glutamine, 1.3 mM CaCl_2_, 2% MEM nonessential amino acids, and 0.2% BSA) for 30 min, washed once, resuspended in 1.3 mL of loading buffer, and warmed to 37 °C in the cuvette. The fluorescence was measured at 340 and 380 nm using a model F-4500 fluorescence spectrophotometer intracellular Ca^2+^ measurement system (Hitachi, Tokyo, Japan), and the background-corrected 340:380 ratio was calibrated.

### 2.9. Measurement of mRNA Expression of Adenosine A3 Receptor

Total RNA was isolated from FcεRI- and c-kit-positive mBMMCs purified with FACS Aria (Becton Dickinson) by using RNeasy Plus Mini according to the manufacturer’s manual. Total RNA was subjected to reverse transcription. The amplification of mAdora3 and GAPDH using cDNA as a template was performed. The following primers were used:

GAPDH forward: 5′-TGACCACAGTCCATGCCATC-3′;
GAPDH reverse: 5′-GACGGACACATTGGGGGTAG-3′;
mAdora forward: 5′-TGTGCTGCTGATCTTCACCC-3′;
mAdora reverse: 5′-AGTGGTAACCGTTCTATATCTGAC-3′.

The reaction products were electrophoresed on a 3% agarose gel containing ethidium bromide. Colonic epithelial cells from BALB/c mice were used as a positive control for the mRNA expression of adenosine A3 receptors.

### 2.10. Statistical Analysis

The data are presented as the mean ± SE. Statistical analyses were performed using a 2-tailed unpaired Student’s *t*-test. Probability (*p*) values < 0.05 were considered statistically significant.

## 3. Results

### 3.1. MMC Hyperplasia and Morphological Interaction between MMCs and Enteric Nerve Fibers in the Colonic Mucosa of FA Mice

**Figure 1:**
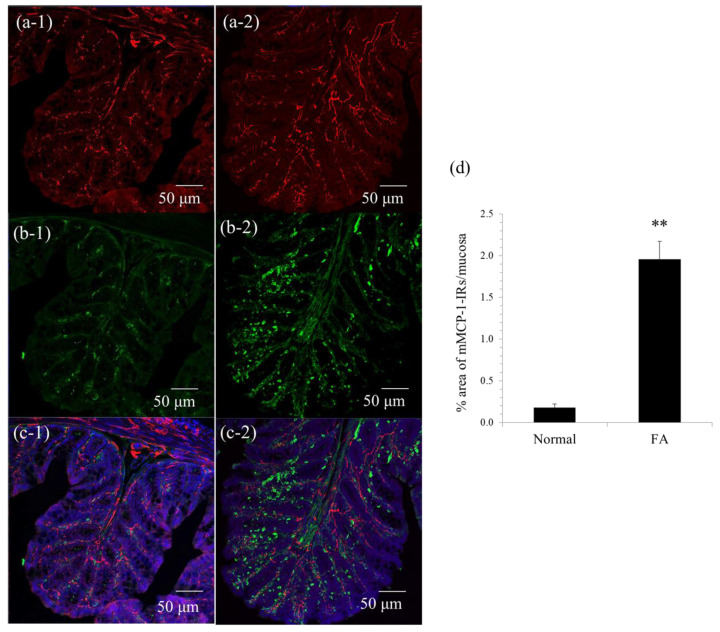
Immunohistochemical analyses of mucosal mast cells (MMCs) and enteric nerve fibers in the proximal colon of normal mice and food allergy (FA) mice. (**a-1**) β3-Tubulin-positive enteric nerve fibers in the colonic mucosa of normal mice. (**a-2**) β3-Ttubulin-positive enteric nerve fibers in the colonic mucosa of FA mice. (**b-1**) Mouse mast cell protease (mMCP) -1-positive mucosal mast cells in the colonic mucosa of normal mice. (**b-2**) mMCP-1-positive mucosal mast cells in the colonic mucosa of FA mice. (**c-1**) A merged view with Figure a-1, b-1, and blue DAPI staining. (**c-2**) A merged view with Figure a-2, b-2, and blue DAPI nuclear staining. (**d**) mMCP-1-immunoreactivities (IRs) markedly increased in the colonic mucosa of FA mice compared to that of normal mice (*n* = 3, ** *p* < 0.01).

**Figure 2:**
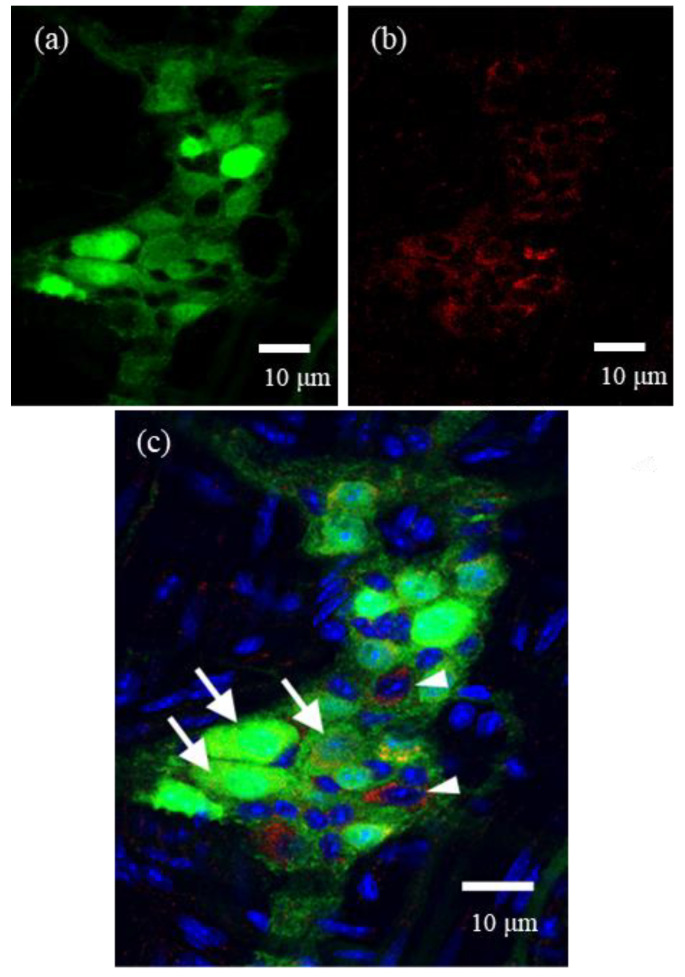
Immunohistochemical analysis of myenteric neurons in a longitudinal muscle myenteric plexus preparation (LMMP) from a normal choline acetyltransferase (ChAT)-eGFP mouse. (**a**) eGFP fluorescence was observed in many myenteric ganglion cells of the ChAT-eGFP mouse (34.1 ± 2.1% of all ganglion cells; all ganglion cells: 112 cells in 5 ganglia from 5 mice). (**b**) FcεRIα immunoreactivities were found in 42.8 ± 4.8% of all ganglion cells. (**c**) Merged view with blue DAPI staining. FcεRI immunoreactivities were detected in cholinergic myenteric neurons (arrows) and noncholinergic ganglion cells (arrowheads). FcεRIα immunoreactivities resided in 68.7 ± 6.8% of ChAT-eGFP-positive neurons (arrows: 26 double-positive neurons/39 ChAT-eGFP-positive neurons in 5 ganglia from 5 mice).

β3-Tubulin-positive enteric nerve fibers were widely distributed in the colonic mucosa of normal mice (Figure 1a-1) and FA mice (Figure 1a-2). As shown in Figure 1b-1, very few mMCP-1-positive MMCs were scattered in the colonic mucosa of normal mice. By contrast, the number of MMCs was dramatically elevated in the colonic mucosa of FA mice (Figure 1b-2). As shown in Figure 1d, MMCs significantly increased approximately 10-fold in the FA mouse colon compared to the normal mouse colon (1.96 ± 0.21% vs. 0.18 ± 0.04%, *n* = 3, ** *p* < 0.01). In addition, we investigated the morphological interaction between MMCs and enteric nerve fibers in the colonic mucosa of FA mice. As a result, we demonstrated that the majority of MMCs were found in close vicinity to enteric nerve fibers in the colonic mucosa of FA mice (Figure 1c-2).

### 3.2. Expression of High-Affinity IgE Receptors on the Mouse Myenteric Plexus

It has been reported that FcεRIs are expressed on mouse SCG neurons and that IgE-antigen stimulation enhances intracellular calcium mobilization in SCG neurons and myenteric ganglionic cells [15]. However, it is still unknown precisely whether FcεRIs are expressed on myenteric neurons, whether myenteric neurons are stimulated by IgE-antigen, and which myenteric neurons respond to IgE-antigen stimulation. Thus, we initially used LMMPs from ChAT-eGFP mice.

As shown in Figure 2a, the strong fluorescence of ChAT-eGFP was highly localized in the cell bodies of myenteric neurons, but weak fluorescence was also present in the nerve fibers. The fluorescence with intensity differences was found in 34.1 ± 2.1% of all ganglion cells (all ganglion cells: 112 cells in five ganglia from five mice). FcεRIα immunoreactivities were observed in 42.8 ± 4.8% of all ganglion cells (five ganglia from five mice, Figure 2b). Furthermore, as shown in the merged view with blue DAPI nuclear staining in Figure 2c, FcεRIα immunoreactivities were detected in noncholinergic ganglion cells (arrowheads) and localized in 68.7 ± 6.8% of ChAT-eGFP-positive neurons (arrows: 26 double-positive neurons/39 ChAT-eGFP-positive neurons in five ganglia from five mice).

### 3.3. Isolated Myenteric Neuron Culture

To investigate precisely whether myenteric neurons, but not other non-neuronal cells in the myenteric ganglion, can be stimulated by IgE-antigen, we attempted to isolate myenteric neurons from the mouse intestine. To verify the successful in vitro culture of isolated myenteric neurons, we, at first, utilized LMMPs from ChAT-eGFP mice and cultured isolated myenteric neurons.

**Figure 3:**
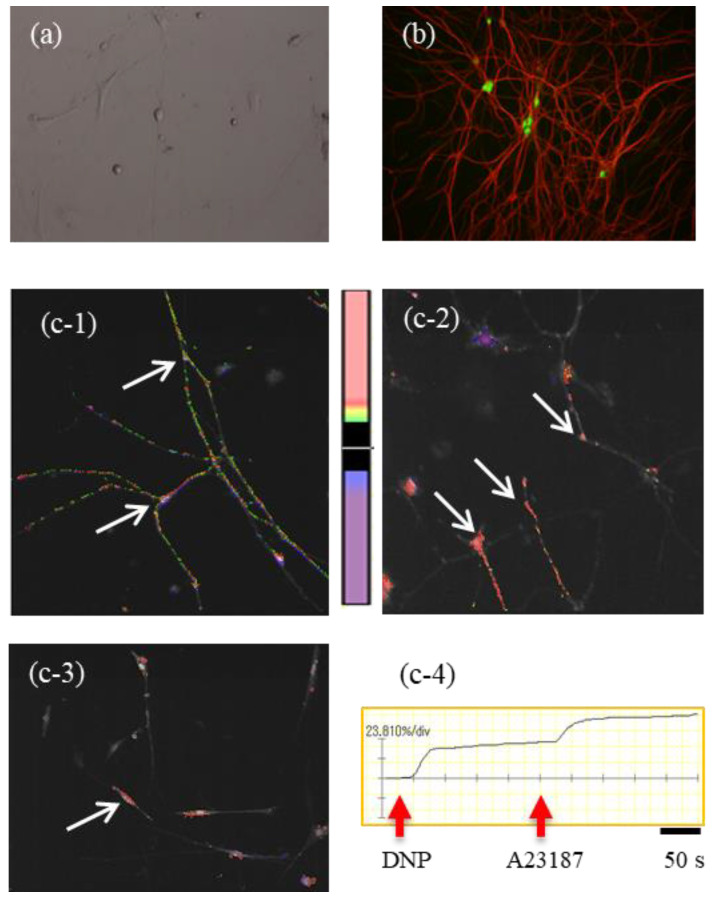
Intracellular calcium mobilization by IgE-antigen stimulation in cultured myenteric neurons. (**a**) In vitro culture of isolated myenteric neurons from a normal ChAT-eGFP mouse on day 6. To verify the successful in vitro culture of myenteric neurons, we utilized LMMPs from a ChAT-eGFP mouse intestine. Several single neuronal cell bodies with axons and several neurites can be seen clearly. (**b**) ChAT-positive myenteric neurons with cell bodies expressing green eGFP fluorescence as well as β3-tubulin-positive axons and neurites expressing red fluorescence from the ChAT-eGFP mouse intestine. (**c-1,c-2,c-3**) Calcium imaging in DNP IgE-stimulated myenteric neurons (arrows). (**c-4**) Representative graph of intracellular calcium mobilization in DNP IgE-stimulated myenteric neurons indicated by the arrow in Figure 3c-3.

As shown in Figure 3a, microscopy observation using the Hoffman Modulation Contrast system showed that isolated myenteric neurons possessed clear cell bodies, axons, and neurites after 6 days in culture. Figure 3b clearly shows the presence of several ChAT-positive myenteric neurons from the intestines of ChAT-eGFP mice with cell bodies expressing green fluorescence as well as β3-tubulin-positive axons and neurites exhibiting red fluorescence. These results clearly indicate that healthy myenteric neurons can be cultured in this in vitro system.

### 3.4. Intracellular Calcium Mobilization by IgE-Antigen Stimulation in Cultured Myenteric Neurons

We previously reported that Th2-prone BALB/c mice, but not Th1-prone C57BL/6 mice, develop FAs in our murine FA model because enhanced Th2-mediated immune responses are responsible for the pathology of our murine FA model [2]. Therefore, to determine whether FcεRIs expressed on myenteric neurons are capable of activating myenteric neurons by IgE-antigen stimulation, the isolated myenteric neurons from BALB/c mice were sensitized with anti-DNP IgE and treated with DNP-BSA. Intracellular calcium mobilization profiles were monitored by the calcium imaging system.

**Figure 4:**
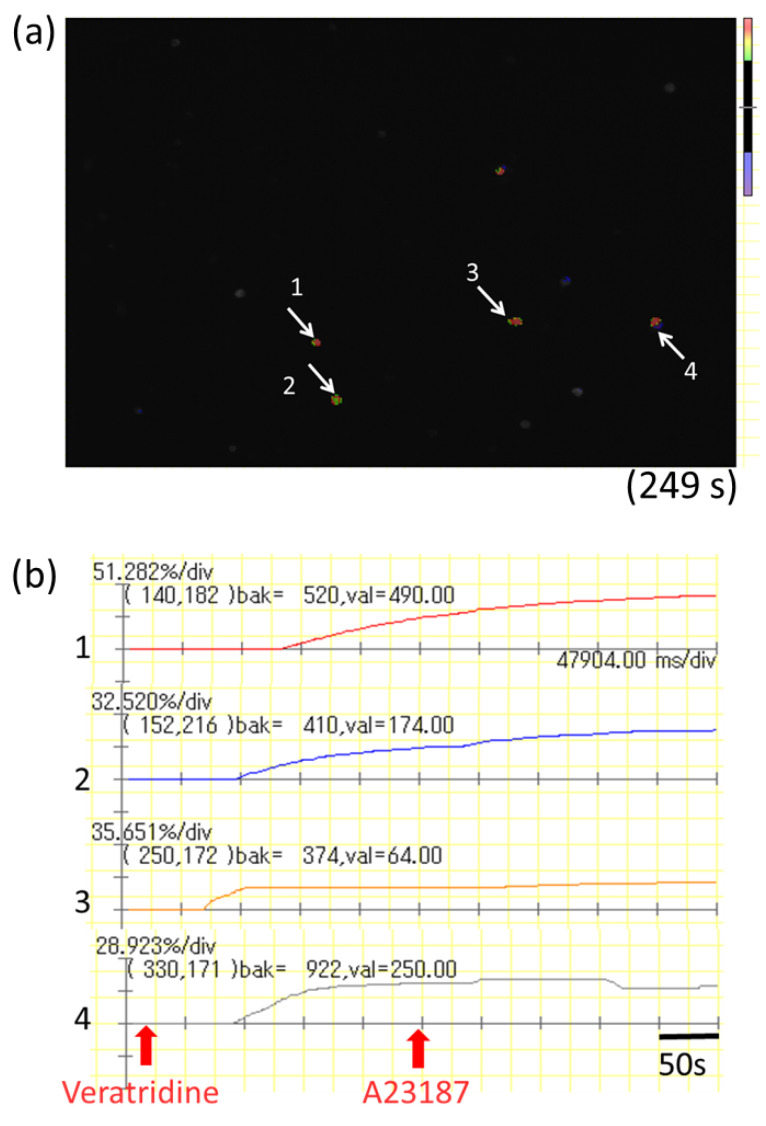
Mucosal-type bone-marrow-derived mast cell (mBMMC) activation induced by neural stimulation of isolated myenteric neurons in the coculture system. (**a**) Isolated myenteric neurons stimulated by neurostimulator veratridine caused obvious increases in intracellular Ca^2+^ concentration in cocultured mBMMCs (arrows). (**b**) Representative graphs of intracellular calcium mobilization are shown. The number of mBMMCs with arrows in Figure 4a corresponds to the number in the graph of intracellular calcium mobilization in Figure 4b.

DNP IgE stimulation increased the [Ca^2+^]i in isolated myenteric neurons (arrows in Figure 3c-1,c-2,c-3). However, not all of the neurons showed increases in the [Ca^2+^]i, and less than 20% of the neurons responded to DNP IgE stimulation. Intracellular calcium mobilization in isolated myenteric neurons shown by the arrow in Figure 3c-3 is illustrated in Figure 3c-4. The mean activation rate in respondent myenteric neurons is 48.1 ± 3.9% (*n* = 30 neurons from eight mice) compared to the calcium ionophore A23187, which was used for the maximum stimulation of isolated myenteric neurons.

### 3.5. Activation of mBMMCs by Bioactive Substances Released from Isolated Myenteric Neurons in Co-Culture System

To investigate whether bioactive substances released from myenteric neurons are capable of activating MMCs under food allergy conditions, isolated myenteric neurons were stimulated by neuron-selective stimulator veratridine in the coculture system of isolated myenteric neurons and mBMMCs. Veratridine stimulation caused pronounced elevation in [Ca^2+^]i in mBMMCs when cocultured with isolated myenteric neurons (arrows in Figure 4a), but not when mBMMCs were cultured alone without these neurons. Intracellular calcium mobilization in mBMMCs, shown by arrows in Figure 4a, is illustrated in Figure 4b. The mean activation rate in respondent mBMMCs is 59.5 ± 2.8% (*n* = 43 from seven mice) of that with A23187, which was used for the maximum stimulation of mBMMCs.

### 3.6. Activation of mBMMCs by Adenosine through Adenosine A3 Receptors

**Figure 5:**
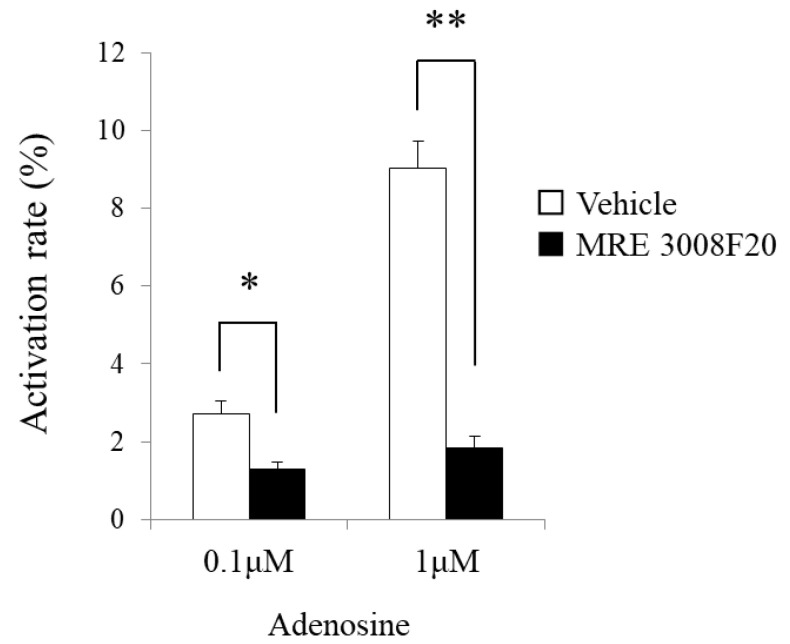
The activation of mBMMCs by adenosine through adenosine A3 receptors. A significant increase in [Ca^2+^]i in mBMMCs resulting from application of 0.1 and 1 μM adenosine (*n* = 3 from 3 mice) was detected using a fluorescence spectrophotometer. Moreover, the elevated [Ca^2+^]i in mBMMCs was significantly suppressed by treatment with the selective adenosine A3 receptor antagonist MRE 308F20 at 10 μM (* *p* < 0.05, ** *p* < 0.01 vs. vehicle, *n* = 3 from three mice).

We investigated whether adenosine activates MMCs with a fluorescence spectrophotometer intracellular Ca^2+^ measurement system. Adenosine markedly increased [Ca^2+^]i in mBMMCs at 0.1 and 1 μM (*n* = 3 from three mice, Figure 5). In addition, elevated [Ca^2+^]i caused by adenosine was significantly reduced by selective adenosine A3 receptor antagonist MRE 3008F20 at 10 μM (adenosine at 0.1 μM: 2.7 ± 0.3% vs. 1.3 ± 0.2%, * *p* < 0.05; adenosine at 1 μM: 9.0 ± 0.7% vs. 1.8 ± 0.3%, ** *p* < 0.01, *n* = 3 from three mice).

### 3.7. Expression of Adenosine A3 Receptors in mBMMCs

**Figure 6:**
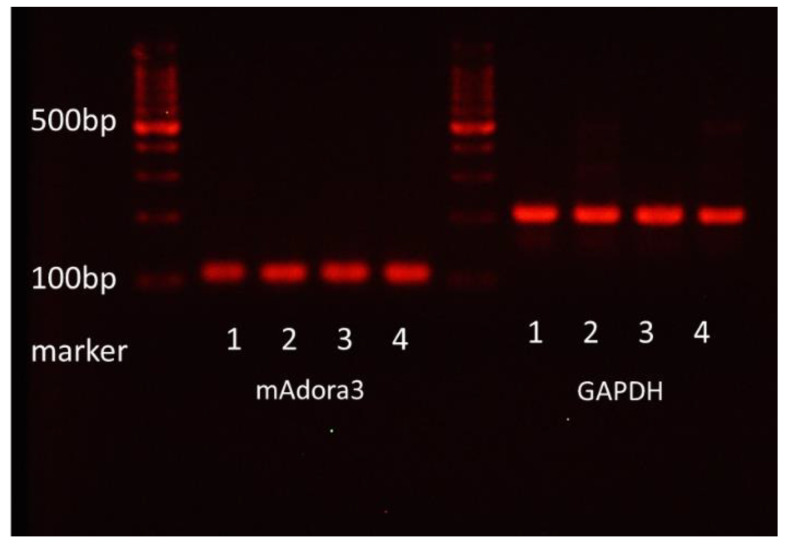
The mRNA expression of adenosine A3 receptors in mBMMCs. The mRNA expression (mAdora3) was detected in the colonic epithelial cells (a positive control; line 1) and mBMMCs after 17 (line 2), 22 (line 3), and 30 days (line 4) of culture using RT-PCR and agarose electrophoresis (a representative result is shown).

BMMCs and LAD2 (human mast cell line) reportedly express adenosine A3 receptors [7], but the expression status of the adenosine A3 receptor gene in MMCs and mBMMCs remains unknown. Thus, we extracted total RNA from mBMMCs after 17, 22, and 30 days of culture and analyzed the mRNA expression of adenosine A3 receptors with PCR and agarose electrophoresis. Consequently, as shown in Figure 6, the mRNA of the adenosine A3 receptor was expressed in mBMMCs.

### 3.8. Suppressive Effect of Adenosine A3 Receptor Antagonist on mBMMC Activation Induced by the Stimulation of Isolated Myenteric Neurons in Co-Culture System

**Figure 7:**
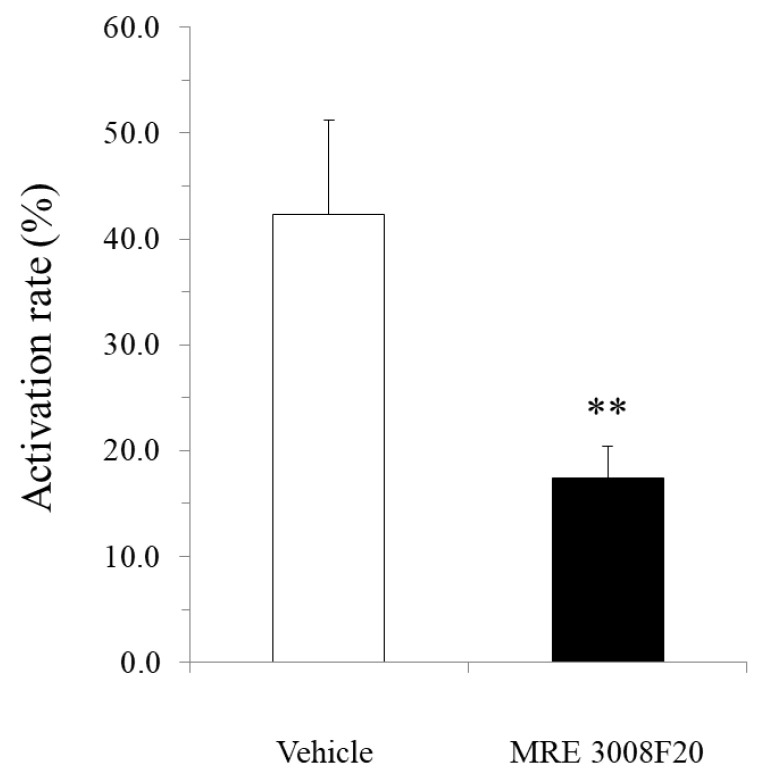
mBMMC activation through adenosine A3 receptors induced by neural stimulation of isolated myenteric neurons in the coculture system. The neural activation of mBMMCs (45.3 ± 6.5%, *n* = 20 from 8 mice) was reduced by adenosine A3 receptor antagonist MRE 3008F20 (10 μM) to 17.5 ± 2.7% (*n* = 26 from 8 mice, ** *p* < 0.01).

To investigate the involvement of adenosine A3 receptors on MMCs in crosstalk between MMCs and myenteric neurons, we applied adenosine A3 receptor antagonist MRE 3008F20 and used veratridine to stimulate only isolated myenteric neurons in the coculture system. Consequently, the activation of isolated myenteric neurons by veratridine led to the enhancement of intracellular calcium mobilization in mBMMCs. As shown in Figure 7, the activation rate stimulated by veratridine in mBMMCc was 45.3 ± 6.5% (*n* = 20 from eight mice), which was suppressed by MRE 3008F20 (10 μM) to 17.5 ± 2.7% (*n* = 26 from eight mice, * *p* < 0.01).

## 4. Discussion

In the present study, we demonstrated that enteric neurons could be activated by IgE-antigen and morphologically and functionally communicate with MMCs through adenosine A3 receptor signaling under FA conditions, suggesting that the neuro-immune crosstalk between enteric neurons and MMCs might be involved in the pathology of FAs.

We demonstrated, for the first time, the expression of FcεRI in myenteric cholinergic neurons in the mouse intestine with immunohistochemistry. In the previous report [15], the study could not focus only on myenteric neurons but examined all cells in the myenteric ganglion, including enteric glial cells, whereas the results of our study clearly revealed elevated [Ca^2+^]i after IgE-antigen stimulation in isolated myenteric neurons. Few studies have documented that neurons express FcεRIs as if they were mast cells and can be directly activated by IgE-antigen stimulation. These studies have mainly employed mouse dorsal root ganglion neurons and SCG neurons [13,14,15]. However, to our knowledge, no report has demonstrated the expression and function of FcεRIs in myenteric neurons.

In both humans and mice, IgE, which is associated with the development of allergies, is usually only present in trace amounts in circulating blood but is greatly increased after the onset of allergies [2]. In contrast to IgE, FcεRIs appear to be constitutively expressed on the myenteric neurons of normal mice. These findings suggest that myenteric neurons can be directly activated by food allergens via FcεRIs, thereby exerting a valid direct role in the pathological mechanisms of FAs. In addition, the gastrointestinal tract is an organ that is prone to immediate allergic reactions, such as allergic diarrhea, which may be attributed to the constitutive expression of FcεRIs on myenteric neurons.

In this study, similarly to our previous reports [2,10], mMCP-1-positive mucosal mast cells multiplied and were juxtaposed with the nerve fibers of enteric neurons in the colonic mucosa of FA mice, which led to the hypothesis that there is pathological crosstalk between them under FA conditions.

Connective tissue mast cells have almost exclusively been reported to be localized in association with the peripheral nervous system, where they are closely aligned, anatomically and functionally, with neurons and neuronal processes throughout the body [24,25,26]. Consequently, the functions of these mast cells are considered to be modulated by neurotransmitters and neuromodulators released from neurons in close proximity, thereby leading to neural involvement in the pathogenesis of mast cell-related diseases. However, little is known about neurotransmitters and neuromodulators involved in the close communication between MMCs and neurons.

Thus, to address the issue under the state of FAs, we established, for the first time, an in vitro coculture system of isolated myenteric neurons and mBMMCs and methods for elucidating the functional crosstalk between isolated myenteric neurons and mBMMCs using a calcium imaging system. Consequently, we revealed that bioactive substances secreted from isolated myenteric neurons activated mBMMCs, indicating the functional crosstalk.

Next, we investigated which neurotransmitters and neuromodulators released from enteric neurons activate MMCs. To date, there are various neurotransmitters and neuromodulators released from enteric neurons, including ACh, purines, autacoids, and neuropeptides. Remarkably, extracellular purines, such as ATP and adenosine, are signaling molecules with a wide range of actions in the nervous system, and extracellular purines play pivotal roles in controlling the chemotaxis, activation, proliferation, and differentiation of immune cells [27,28].

Purinergic P1 receptors are activated by endogenous adenosine, but whether adenosine is stored in synaptic vesicles and released as a classical neurotransmitter is unclear. One way adenosine may be made available in the extracellular space is from the breakdown of ATP released as a neurotransmitter. Thus, adenosine is released indirectly by the hydrolysis of released ATP through the extracellular enzyme, ectonucleotidase pathway. However, BMMCs highly express ectonucleotidase CD39, but not ecto-5′-nucleotidase (CD73) and, hence, are unable to directly convert ATP to adenosine [22]. In addition, a study in the ENS failed to show any appreciable effect when the breakdown of ATP was prevented [29]. Thus, it is suggested that this route for adenosine formation, although plausible, may not be physiologically relevant in the crosstalk between enteric neurons and mast cells.

In addition to the extracellular production of adenosine through nucleotide hydrolysis, adenosine can be produced in the cytosol of neurons as a consequence of the metabolic process and released directly into the extracellular space as adenosine through nucleoside transporters [27,30]. The electrical stimulation of LMMPs reportedly increases the outflow of adenosine, inosine and hypoxanthine [29].

Of the four subtypes of adenosine receptor, adenosine A2b receptors and A3 receptors are known to be associated with the elevation of intracellular calcium concentration. It has been reported that adenosine A3 receptors on mast cells are involved in the induction of inflammation [6,7]. Hua et al. demonstrated that adenosine A3 receptor-deficient mice and mast cell-deficient mice are resistant to adenosine-induced airway hyperresponsiveness and that the reconstitution of mast cell-deficient mice with wild-type mast cells restored adenosine-induced airway hyperresponsiveness, whereas reconstitution with adenosine A3 receptor-deficient mast cells failed to restore it [31]. Thus, it has been suggested that mast cells are deeply associated with the pathogenesis of airway inflammation via adenosine A3 receptors.

In the present study, we investigated the gene expression of adenosine A3 receptors in mBMMCs and revealed that adenosine A3 receptors were highly expressed. In addition, mBMMCs were activated by exogenous adenosine, which was reversed by adenosine A3 receptor antagonist MRE 3008F20. Furthermore, using the coculture system of isolated myenteric neurons and mBMMCs, we showed that veratridine-induced activation in mBMMCs was significantly suppressed by adenosine A3 receptor antagonist MRE 3008F20, indicating that adenosine is involved in the functional interaction between isolated myenteric neurons and mBMMCs through adenosine A3 receptor signaling.

**Figure 8:**
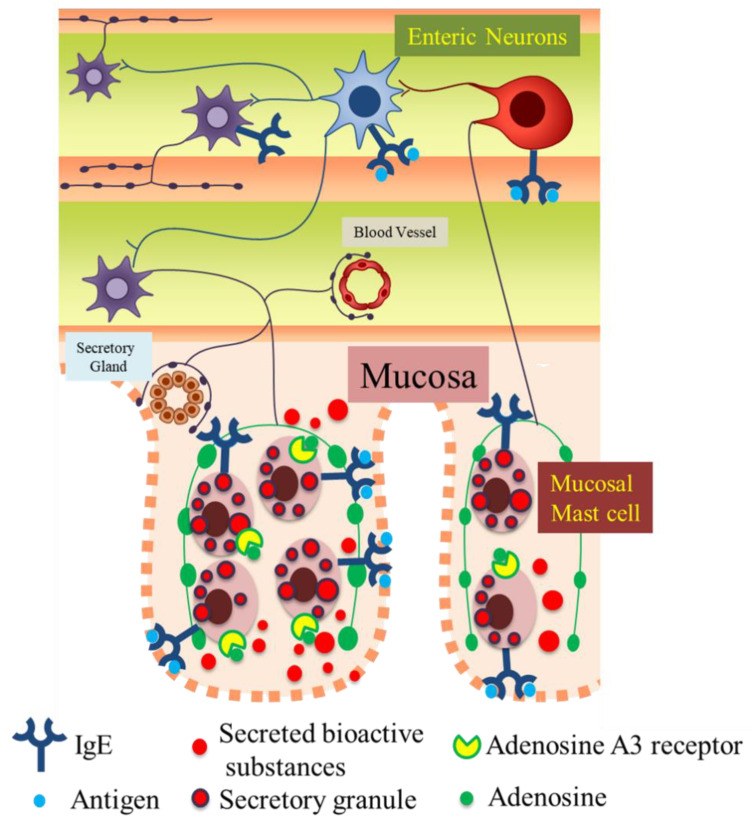
Graphical abstract. Our findings and conclusions from this study using a mouse model are as follows. IgE-antigen stimulates FcεRIs on not only mucosal mast cells but also enteric neurons and sequentially activates the excitatory neural circuitry in the enteric nervous system under the state of a FA. Furthermore, activated enteric neurons release adenosine and further activate mucosal mast cells by neuro-immune interactions through adenosine A3 receptors on mucosal mast cells, resulting in excessive activation of MMCs, thereby aggravating the FA.

The precise pathophysiological role of enteric neurons in intestinal immune diseases, including FAs, has not been elucidated. In FA pathology, the blood IgE level is usually elevated. Thus, it is indicated that IgE-antigen stimulates FcεRIs on not only MMCs but also enteric neurons and sequentially activates the excitatory neural circuitry in the enteric nervous system, which as one consequence, results in the appearance of allergic symptoms, such as food allergic diarrhea. Moreover, activated enteric neurons further activate MMCs by neuro-immune interactions through adenosine A3 receptors, resulting in excessive activation of MMCs, thereby inducing an excessive release of inflammatory chemical mediators and contributing to the pathological process in FAs (Figure 8). In addition, the excessive secretion of various bioactive substances from MMCs induces the disruption of barrier functions of the mucosal epithelium and increases the permeability of the epithelial layer in the intestine [32], resulting in the aggravation of FAs.

Therefore, in conclusion, our findings derived from a murine model indicate that FcεRI-activated myenteric neurons play a pathological role in further exacerbating the pathology of food allergy through their interaction with MMCs.

## Data Availability

This study did not report any data.
